# Light‐Promoted Hydrazine Dehydrogenation over Ni/NH_2_‐MIL‐125: Unraveling Mechanisms for Efficient Hydrogen Production

**DOI:** 10.1002/advs.202519320

**Published:** 2025-12-05

**Authors:** Jianjun Long, Caili Hu, Qilu Yao, Kun Wang, Gang Feng, Shaowen Cao, Zhang‐Hui Lu

**Affiliations:** ^1^ Key Laboratory of Green Catalysis of Jiangxi Education Institutes Key Laboratory of Green Hydrogen and Advanced Catalysis of Jiangxi Province Key Laboratory of Fluorine and Silicon for Energy Materials and Chemistry of Ministry of Education College of Chemistry and Materials Jiangxi Normal University Nanchang 330022 China; ^2^ Key Laboratory for Environment and Energy Catalysis of Jiangxi Province College of Chemistry and Chemical Engineering Nanchang University Nanchang 330031 China; ^3^ State Key Laboratory of Advanced Technology for Materials Synthesis and Processing Wuhan University of Technology Wuhan 430070 China

**Keywords:** dehydrogenation, hydrous hydrazine, light‐promoted, NH_2_‐MIL‐125(Ti), Nickel

## Abstract

Developing efficient, low‐cost, and durable catalysts for the complete dehydrogenation of hydrous hydrazine (N_2_H_4_·H_2_O) remains a significant challenge. Herein, a high‐performance, visible‐light‐responsive Ni‐loaded amino‐functionalized MIL‐125 (Ni/NH_2_‐MIL‐125) catalyst is fabricated via a facile chemical reduction method, which, for the first time, enables visible‐light‐enhanced N_2_H_4_ dehydrogenation using a non‐precious metal catalyst. Under visible light irradiation, Ni/NH_2_‐MIL‐125 achieves a turnover frequency (TOF) of 220.2 h^−1^ at 323 K, more than twice that in the dark (108.1 h^−1^) and outperforms most reported non‐precious metal catalysts. Characterizations and theoretical calculations reveal that the enhanced light‐promoted activity arises from light‐induced electron transfer from the NH_2_‐MIL‐125 to the Ni active sites, which synergistically strengthens N_2_H_4_ adsorption and lowers the energy barrier for N─H bond cleavage, the rate‐determining step. This work establishes visible‐light promotion as a powerful strategy to enhance non‐precious metal catalysis and offers a promising avenue for nitrogen‐based hydrogen storage systems.

## Graphical abstract

Highly dispersed and electron‐rich Ni nanoparticles (≈2.7 nm) supported on NH_2_‐MIL‐125 significantly promote the dehydrogenation of hydrous hydrazine under visible‐light irradiation, achieving a turnover frequency of 220.2 h^−1^ at 323 K, which is more than twice that in the dark (108.1 h^−1^) and outperforms most reported non‐precious metal catalysts.

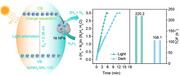

## 1 Introduction

The extensive use of fossil fuels has caused serious environmental challenges, including atmospheric pollution and global warming, highlighting the urgent need for clean and sustainable energy alternatives.^[^ ^1^, ^2^, ^3^, ^4^ ^]^ Hydrogen, characterized by its zero‐emission nature, high energy density, and abundant availability, stands out as a promising renewable energy carrier.^[^ ^5^, ^6^, ^7^ ^]^ However, large‐scale implementation of hydrogen energy faces significant technical hurdles related to safe and efficient storage and transportation.^[^ ^8^ ^]^ Chemical hydrogen storage, which involves confining hydrogen within the stable chemical bonds of specific compounds, offers enhanced safety and efficiency in hydrogen handling, owing to the intrinsic stability and high volumetric density of these materials.^[^ ^9^, ^10^, ^11^ ^]^ Among various chemical hydrogen carriers, hydrous hydrazine (N_2_H_4_·H_2_O) is particularly attractive due to its high gravimetric hydrogen content (8.0 wt.%), ambient‐temperature liquid storage, and the potential for clean decomposition into only H_2_ and N_2_ (Equation 1).^[^ ^12^, ^13^, ^14^, ^15^, ^16^ ^]^ Specifically, hydrous hydrazine shows significant potential as a hydrogen storage material for specialized applications, such as unmanned space vehicles and submarine power systems, where hydrazine is commonly utilized as a propellant. Furthermore, the potential for reversible regeneration through nitrogen hydrogenation further underscores its practical potential.^[^ ^17^, ^18^, ^19^, ^20^, ^21^, ^22^, ^23^ ^]^ Nevertheless, catalytic decomposition of N_2_H_4_ often suffers from the undesired formation of NH_3_ (Equation 2), which irreversibly poisons fuel cell catalysts and severely limits efficient hydrogen utilization.^[^ ^24^, ^25^, ^26^, ^27^, ^28^, ^29^, ^30^, ^31^ ^]^ Therefore, developing catalysts that simultaneously deliver high activity and complete hydrogen selectivity (100% H_2_) is of paramount importance.

(1) $$N_{2}H_{4}\left(l\right)\to N_{2}\left(g\right)+2H_{2}\left(g\right)$$

(2) $$3N_{2}H_{4}\left(l\right)\to 4NH_{3}\left(g\right)+N_{2}\left(g\right)$$

To date, noble metal‐modified Ni‐based catalysts (e.g., NiPt, NiRh, NiIr, NiPd) have achieved exceptional activity with perfect H_2_ selectivity (100%). However, their reliance on scarce and expensive precious metals severely limits large‐scale industrial adoption.^[^ ^32^, ^33^, ^34^, ^35^, ^36^ ^]^ Consequently, significant research efforts have focused on non‐precious metal alternatives (e.g., Ni, NiCo, NiFe, NiCu).^[^ ^37^, ^38^, ^39^, ^40^ ^]^ Yet, these typically suffer from low activity, insufficient hydrogen selectivity, and poor stability, leaving the development of efficient and robust non‐precious metal catalysts as a pressing challenge.

Visible light irradiation has recently emerged as a powerful strategy to boost the catalytic activity of non‐precious metals in various heterogeneous dehydrogenation reactions,^[^ ^41^, ^42^, ^43^ ^]^ such as formic acid (HCOOH),^[^ ^44^ ^]^ ammonia borane (NH_3_BH_3_),^[^ ^45^ ^]^ and sodium borohydride (NaBH_4_).^[^ ^46^ ^]^ Inspired by these advances, we aimed to introduce light‐assisted catalysis into N_2_H_4_·H_2_O dehydrogenation, a reaction that proceeds sluggishly when catalyzed by non‐precious metal catalysts and has not yet been explored in a photocatalytic context. For non‐plasmonic metals such as Ni, visible light can induce interband transitions that increase the electron density,^[^ ^47^, ^48^, ^49^, ^50^ ^]^ yet this effect alone is typically insufficient to significantly enhance catalytic performance.^[^ ^51^ ^]^ To overcome this limitation, photosensitizing supports capable of generating and transferring additional photogenerated electrons are required. Metal‐organic frameworks (MOFs) functionalized with amine or porphyrin groups are particularly promising candidates. Under visible light, their organic linkers in these MOFs undergo electronic excitation from the highest occupied molecular orbital to the lowest unoccupied molecular orbital, producing photogenerated electrons that can migrate to supported metal nanoparticles (NPs) and enrich their electron density.^[^ ^52^ ^,^ ^53^ ^]^ Beyond light‐harvesting, MOFs also possess ordered and tunable structures that enable precise modulation of electronic structure, stabilize metal NPs, and provide a platform for elucidating structure–activity relationships in photocatalysis. Integrating non‐plasmonic Ni NPs with a photoactive MOF support is therefore expected to synergistically increase the electron density at Ni active sites under visible light irradiation, potentially overcoming the kinetic limitations of N_2_H_4_ dehydrogenation and achieving a breakthrough in catalytic performance.

Herein, we report for the first time the synthesis of a visible‐light‐responsive non‐precious metal catalyst for N_2_H_4_ dehydrogenation, achieved by immobilizing Ni NPs on NH_2_‐MIL‐125 framework via a simple chemical reduction method. Characterizations and theoretical calculations reveal that NH_2_‐MIL‐125 not only stabilizes and disperses Ni NPs but also acts as an efficient photosensitizer, generating photogenerated electrons under visible light and transferring them to Ni active sites. This enhances the electron density of Ni NPs, promoting N─H bond cleavage, the rate‐determining step. As a result, under visible light, Ni/NH_2_‐MIL‐125 catalyzes the dehydrogenation of N_2_H_4_·H_2_O with a TOF of 220.2 h^−1^ at 323 K, twice that achieved in the dark (108.1 h^−1^) and surpassing most non‐precious metal catalysts reported. This work offers a new strategy for designing high‐performance N_2_H_4_ dehydrogenation catalysts for chemical hydrogen storage.

## 2 Results and Discussion

**Figure 1:**
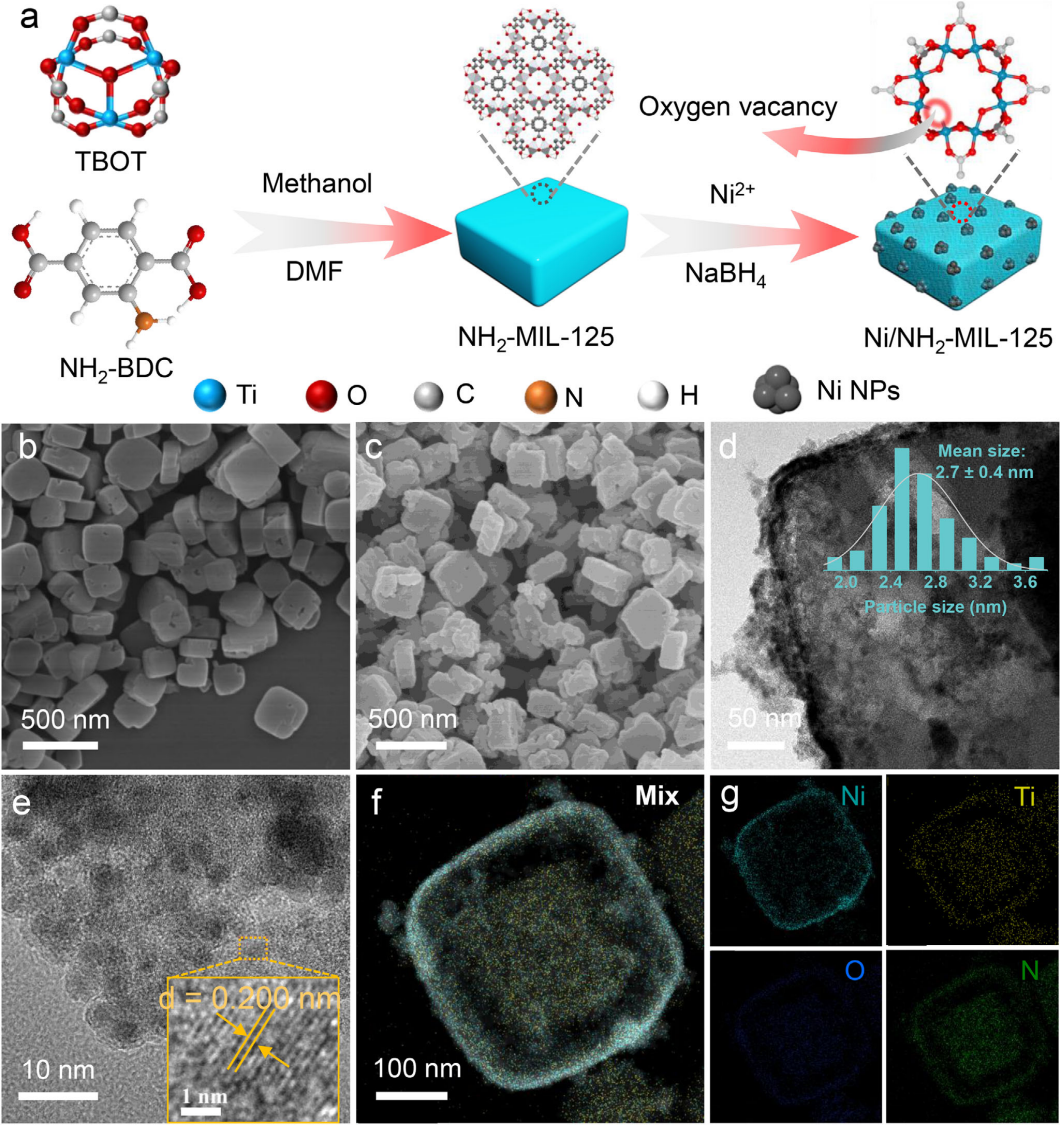
a) Schematic diagram for the preparation of Ni/NH_2_‐MIL‐125; SEM images of b) NH_2_‐MIL‐125 and c) Ni/NH_2_‐MIL‐125; d) TEM image and (inset of d) particle size distribution of Ni/NH_2_‐MIL‐125; e) HRTEM image, f) HAADF‐STEM image, and g) the corresponding EDX mapping images of Ni/NH_2_‐MIL‐125.

The Ni/NH_2_‐MIL‐125 catalyst was synthesized via a chemical reduction method (**Figure** **1**a). First, NH_2_‐MIL‐125(Ti) was obtained via a solvothermal method using titanium butoxide (TBOT) and 2‐amino‐1,4‐benzenedicarboxylic acid (NH_2_‐BDC) as precursors. The as‐prepared MOF was then dispersed with the Ni precursor in deionized water under ultrasonic treatment to achieve a uniform suspension. Subsequently, NaBH_4_ was added as the reducing agent under continuous stirring. Following the reduction process, the Ni/NH_2_‐MIL‐125 catalyst was collected by centrifugation, washed with deionized water and ethanol, and vacuum‐dried for further characterization.

Scanning electron microscopy (SEM) image (Figure 1b) displays that the pristine NH_2_‐MIL‐125 exhibits a well‐defined disc structure with particle sizes ranging from 200–300 nm. After Ni loading, the surface of the NH_2_‐MIL‐125 becomes noticeably rougher (Figure 1c), indicating successful deposition of Ni NPs onto the MOF framework. To gain further insights into the microstructure of the synthesized catalyst, transmission electron microscopy (TEM) was performed. As shown in Figure 1d,e, Ni NPs with an average diameter of ≈2.7 nm are homogeneously distributed on the surface of the NH_2_‐MIL‐125 support. The high‐resolution TEM (HRTEM) image (Figure 1e) shows distinct lattice fringes with a lattice spacing of 0.200 nm, corresponding to the (111) plane of metallic Ni (JCPDS no. 88–2326), further verifying successful immobilization of Ni NPs. Further structural and compositional analyses were carried out using high‐angle annular dark‐field scanning TEM (HAADF‐STEM) (Figure 1f) combined with energy‐dispersive X‐ray spectroscopy (EDS) elemental mapping (Figure 1g) and EDX pattern (Figure S1, Supporting Information), which collectively confirm the presence and uniform distribution of Ni, Ti, O, C, and N elements across the entire Ni/NH_2_‐MIL‐125 catalyst. In contrast, unsupported Ni NPs (Figure S2, Supporting Information) are heavily agglomerated and have a much larger particle size (11.6 nm), highlighting the critical role of NH_2_‐MIL‐125 in dispersing Ni, suppressing aggregation, and creating abundant accessible active sites.

**Figure 2:**
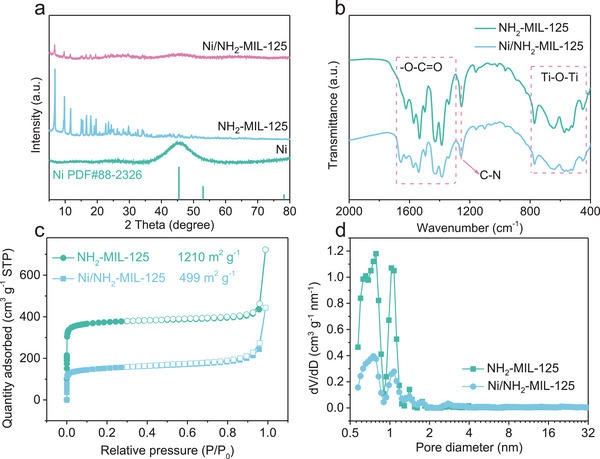
a) XRD patterns of Ni, NH_2_‐MIL‐125, and Ni/NH_2_‐MIL‐125; b) FT‐IR spectra, c) N_2_ adsorption/desorption, and d) pore size distribution of NH_2_‐MIL‐125 and Ni/NH_2_‐MIL‐125.

X‐ray diffraction (XRD) patterns (**Figure** **2**a) were collected to analyze the crystallinity and phase composition of Ni, NH_2_‐MIL‐125, and Ni/NH_2_‐MIL‐125. The pristine NH_2_‐MIL‐125 displays characteristic diffraction peaks consistent with literature reports,^[^ ^54^ ^]^ confirming the successful synthesis. After Ni incorporation, these characteristic peaks are still clearly retained in Ni/NH_2_‐MIL‐125, indicating preservation of the crystalline MOF framework. However, the peak intensities are significantly decreased compared to pristine NH_2_‐MIL‐125, which is mainly due to the partial reduction of Ti^4+^ to Ti^3+^ by NaBH_4_ during Ni^2+^ reduction. This process generates oxygen vacancies and increases lattice disorder, thereby lowering the crystallinity.^[^ ^55^ ^]^ To verify this, a series of Ni/NH_2_‐MIL‐125 samples was synthesized using different amounts of NaBH_4_. As shown in Figure S3a (Supporting Information), the XRD peak intensities of Ni/NH_2_‐MIL‐125 progressively decrease with increasing NaBH_4_ dosage, confirming that the structural disorder correlates with the extent of Ti^4+^ to Ti^3+^ reduction. This observation is further supported by the electron paramagnetic resonance (EPR) (Figure S3b, Supporting Information), which shows increased concentrations of oxygen vacancies. For unsupported Ni NPs, a broad diffraction peak near 45.5° is observed, corresponding to the (111) plane of metallic Ni (JCPDS No. 88–2326). However, this peak is very weak in Ni/NH_2_‐MIL‐125, suggesting that Ni NPs are highly dispersed, ultrafine in size (2.7 nm), and possess low crystallinity, consistent with TEM results. The poor crystallinity and high dispersion of Ni NPs are expected to expose more active sites, which may contribute positively to the catalytic performance.

Fourier transform infrared (FTIR) spectroscopy was employed to investigate the chemical structure of NH_2_‐MIL‐125 and Ni/NH_2_‐MIL‐125 (Figure 2b). The FTIR spectrum of pristine NH_2_‐MIL‐125 exhibits characteristic absorption bands in the 1300–1600 cm^−1^ region, attributed to the asymmetric and symmetric stretching vibrations of carboxylate groups. Additional peaks observed at 1258 cm^−1^ and in the range of 400–800 cm^−1^ correspond to the C─N stretching and Ti─O─Ti vibrations, respectively,^[^ ^56^ ^]^ confirming the integrity of the MOF framework. After Ni incorporation, the FTIR profile of the composite remains largely unchanged, indicating that the coordination environment and the backbone of the NH_2_‐MIL‐125 are well preserved. This suggests that the incorporation of Ni NPs does not significantly disrupt the molecular structure of the MOF support.

Nitrogen adsorption–desorption measurements were carried out to evaluate the textural properties of NH_2_‐MIL‐125 and Ni/NH_2_‐MIL‐125. As shown in Figure 2c,d, both materials exhibit typical type I isotherms, characteristic of microporous frameworks. The pristine NH_2_‐MIL‐125 displays a high BET surface area of 1210 m^2^ g^−1^ and a total pore volume of 1.12 cm^3^ g^−1^. After Ni loading, the surface area of Ni/NH_2_‐MIL‐125 markedly decreases to 499 m^2^ g^−1^, along with a reduction in pore volume to 0.69 cm^3^ g^−1^ and a slight increase in average pore diameter. These changes are primarily attributed to the blockage of partial MOF pores by the deposited Ni NPs and the localized structural collapse during the reduction process, which concurrently verifies the successful anchoring of Ni active sites onto the MOF support surface. In addition, a magnified view of the overlapping region of the N_2_ adsorption/desorption isotherms (Figure S4, Supporting Information) exhibits slight hysteresis loops, indicating the existence of minor mesopores in NH_2_‐MIL‐125 and Ni/NH_2_‐MIL‐125. After Ni loading, the hysteresis loop becomes slightly more pronounced, which may be attributed to additional structural defects generated during Ni incorporation and subsequent reduction.

**Figure 3:**
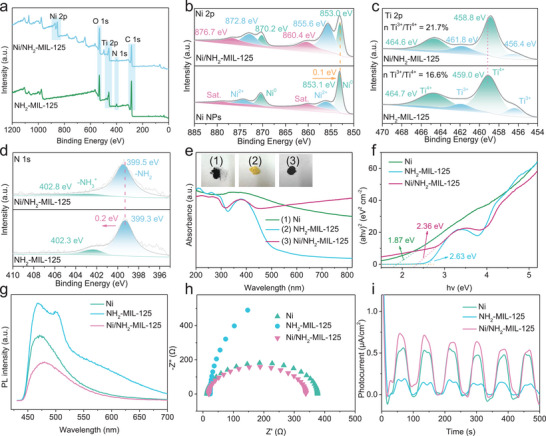
a) Survey XPS, b) Ni 2p, c) Ti 2p, and d) N 1s spectra of Ni/NH_2_‐MIL‐125; e) UV–Vis diffuse reflectance spectroscopy, f) Tauc plots, g) PL spectra, h) EIS, and i) Transient photocurrent response of Ni, NH_2_‐MIL‐125, and Ni/NH_2_‐MIL‐125.

X‐ray photoelectron spectroscopy (XPS) was further conducted to examine the elemental composition and chemical states of the synthesized Ni/NH_2_‐MIL‐125. The survey XPS spectrum shows clear signals for Ni, Ti, C, N, and O elements (**Figure** **3**a), in good agreement with the EDX results (Figure S1, Supporting Information). In the high‐resolution Ni 2p spectrum of Ni/NH_2_‐MIL‐125 (Figure 3b), the peaks at 853.0 and 870.2 eV are assigned to Ni^0^ 2p_3/2_ and Ni^0^ 2p_1/2_, respectively. The peaks at 855.6 and 872.8 eV correspond to Ni^2+^ 2p_3/2_ and Ni^2+^ 2p_1/2_, primarily due to partial oxidation during sample preparation.^[^ ^57^ ^]^ Depth‐profiling XPS (Figure S5, Supporting Information) confirms that Ni^2+^ signal originates only from a thin surface layer. With increasing Ar⁺ etching time, the Ni^0^ signal becomes progressively stronger while the Ni^2+^ signal feature diminishes and nearly disappears after 120 s, indicating that the bulk Ni remains metallic. Satellite peaks at 860.4 and 876.7 eV are also observed. The Ti 2p spectrum of Ni/NH_2_‐MIL‐125 (Figure 3c) shows Ti^4+^ signals at 458.8 and 464.6 eV and Ti^3+^ signals at 456.4 and 461.8 eV, corresponding to a Ti^3+^/Ti^4+^ ratio of 21.7%. The formation of Ti^3+^ and oxygen vacancies (Ov) originates from the partial reduction of Ti^4+^ during NaBH_4_ treatment, as further supported by O 1s analysis (Figure S6, Supporting Information), where the Ov proportion increases from 16.7% in NH_2_‐MIL‐125 to 28.9% after Ni loading. The N 1s spectra (Figure 3d) of NH_2_‐MIL‐125 and Ni/NH_2_‐MIL‐125 each show two peaks corresponding to the ─NH_2_ and ─NH_3_^+^ species, confirming the preservation of amino linkers after Ni incorporation. In Ni/NH_2_‐MIL‐125, these peaks shift slightly to higher binding energies (399.5 and 402.8 eV) compared with those in NH_2_‐MIL‐125 (399.3 and 402.3 eV), indicating electron donation from the amino‐containing framework to the metallic Ni. Consistently, the binding energies of Ni^0^ in Ni/NH_2_‐MIL‐125 (853.0 and 870.2 eV) are lower than those in unsupported Ni NPs (853.1 and 870.4 eV), further confirming some electron transfer from MOF to Ni. Collectively, the XPS results demonstrate that Ni loading enhances the Ti^3+^/Ov defects concentration and promotes electron transfer from NH_2_‐MIL‐125 to Ni, generating electron‐rich Ni sites that are highly favorable for N_2_H_4_ dehydrogenation.

The light absorption properties of Ni NPs, NH_2_‐MIL‐125, and Ni/NH_2_‐MIL‐125 were characterized by UV–Vis diffuse reflectance spectroscopy. As shown in Figure 3e, the pristine NH_2_‐MIL‐125 exhibits low light absorption capacity at wavelengths greater than 500 nm. In contrast, the catalyst modified with Ni NPs exhibits a substantially enhanced light‐harvesting ability across the entire measured wavelength range (200‐800 nm), highlighting its advantage for solar energy utilization. The Tauc plots derived from the absorption spectra (Figure 3f) reveal that the bandgap energies of Ni NPs, NH_2_‐MIL‐125, and Ni/NH_2_‐MIL‐125 are 1.87, 2.63, and 2.36 eV, respectively. These results indicate that the enhanced light absorption capacity of Ni/NH_2_‐MIL‐125 originates from the reduced band gap induced by the introduction of Ni NPs. To further investigate the carrier separation efficiency, photoluminescence (PL) spectroscopy was conducted. As depicted in Figure 3g, all three samples exhibit their strongest emission signals ≈470 nm. Notably, the PL intensity of Ni/NH_2_‐MIL‐125 is significantly lower than that of free Ni and NH_2_‐MIL‐125, indicating suppressed recombination of photogenerated charge carriers. This quenching effect likely originates from the synergistic interaction between Ni and NH_2_‐MIL‐125. Figure 3h presents the electrochemical impedance spectra (EIS) of the above three samples. In Nyquist plots, the arc radius corresponds to the charge transfer resistance, with a smaller radius indicating lower interfacial resistance. The EIS results confirm that the Ni/NH_2_‐MIL‐125 possesses markedly reduced charge transfer resistance compared to its individual components. Figure 3i displays the transient photocurrent responses under intermittent illumination and dark conditions. The intensity of photocurrent is a key indicator for evaluating the separation efficiency of photogenerated electron‐hole pairs, as it directly reflects the light energy conversion into electrical current by the material. The enhanced photocurrent response implies more efficient carrier separation, migration, and transfer, thereby improving photocatalytic performance. Importantly, under identical illumination conditions, Ni/NH_2_‐MIL‐125 exhibits the highest photocurrent density, confirming that the synergistic coupling between Ni and NH_2_‐MIL‐125 effectively facilitates the separation of photogenerated charge carriers. Moreover, comparison with MIL‐125 and Ni/MIL‐125 shows that the introduction of amino groups further narrows the bandgap (Figure S7a, Supporting Information), reduces electrochemical impedance (Figure S7b, Supporting Information), enhances photocurrent density (Figure S7c, Supporting Information), and promotes the separation and transfer of photogenerated electron‐hole pairs (Figure S7d, Supporting Information), thereby facilitating the formation of electron‐rich Ni sites. These combined effects ultimately account for the superior catalytic activity of Ni/NH_2_‐MIL‐125.

**Figure 4:**
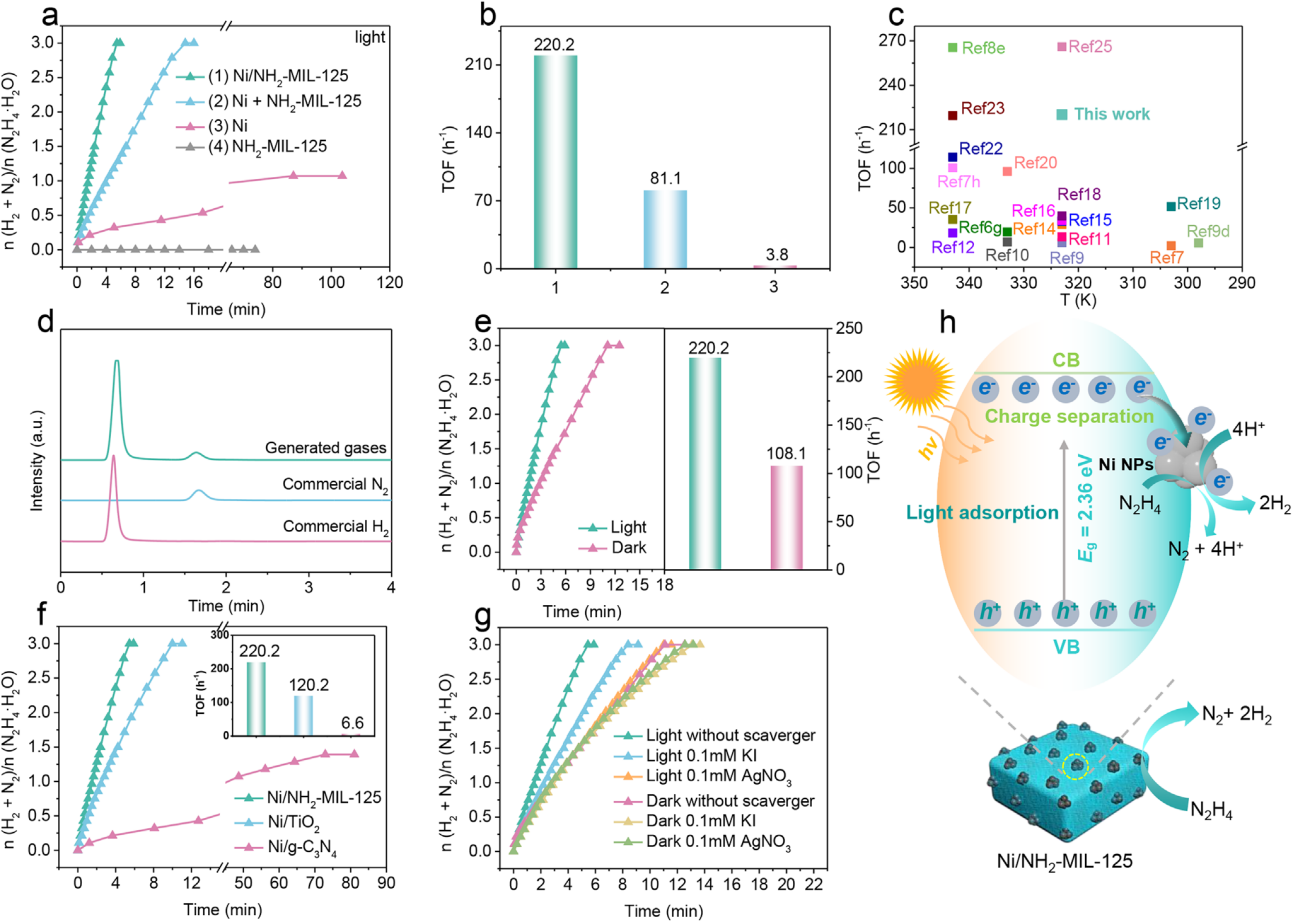
a) Time course plots for hydrogen evolution from aqueous N_2_H_4_ solution 0.4 m, 5 mL) over Ni/NH_2_‐MIL‐125, Ni NPs, NH_2_‐MIL‐125, and Ni + NH_2_‐MIL‐125; b) The corresponding the TOF values; c) Comparison of the catalytic activities for N_2_H_4_ dehydrogenation with previously reported non‐precious catalysts; d) GC‐TCD spectra of commercial H_2_, commercial N_2_, and generated gases from aqueous N_2_H_4_ solution catalyzed by Ni/NH_2_‐MIL‐125; Time course plots for hydrogen evolution from aqueous N_2_H_4_ solution over e) Ni/NH_2_‐MIL‐125 in the dark and under visible light irradiation, f) Ni/NH_2_‐MIL‐125, Ni/TiO_2_, and Ni/g‐C_3_N_4_ under visible light irradiation, and g) Ni/NH_2_‐MIL‐125 with/without charge scavengers; h) The plausible mechanism of N_2_H_4_ dehydrogenation catalyzed by Ni/NH_2_‐MIL‐125 under visible light irradiation.

The catalytic activity of Ni/NH_2_‐MIL‐125 and various reference samples toward N_2_H_4_ dehydrogenation was systematically evaluated using a standard gas collection setup under visible light irradiation. As illustrated in **Figure** **4**a,b, the Ni/NH_2_‐MIL‐125 shows an outstanding performance, achieving complete dehydrogenation of N_2_H_4_ within only 5.45 min and delivering a high TOF of 220.2 h^−1^ at 323 K. This performance surpasses that of most previously reported non‐precious catalysts (Figure 4c and Table S1, Supporting Information). By comparison, unsupported Ni NPs show limited catalytic activity, with a low H_2_ selectivity of 28% and a TOF of only 3.8 h^−1^, releasing 1.07 equivalents of N_2_ and H_2_ within 87 min under the identical conditions. Notably, only NH_2_‐MIL‐125 (Figure 4a), commercial Ni particles (≈44 µm) (Figure S8, Supporting Information), or NiO (Figure S9, Supporting Information) exhibit no catalytic activity, confirming that nanosized metallic Ni serves as the active center. In addition, to further elucidate the synergy between Ni and NH_2_‐MIL‐125, a physical mixture of bare Ni NPs and NH_2_‐MIL‐125 (denoted as Ni + NH_2_‐MIL‐125) was prepared and evaluated. Although this physical mixture also enabled complete dehydrogenation of N_2_H_4_, its catalytic activity (81.1 h^−1^) is markedly inferior to that of the Ni/NH_2_‐MIL‐125. Additionally, Ni/MIL‐125 (140.5 h^−1^) also shows lower catalytic activity than Ni/NH_2_‐MIL‐125 (Figure S10, Supporting Information). These results demonstrate that the strong interactions between Ni and NH_2_‐MIL‐125 are crucial for the catalytic performance, and that the ─NH_2_ functional groups play an important role in enhancing the performance. In addition, to investigate whether the catalyst undergoes self‐decomposition due to its intrinsic catalytic activity, control experiments were conducted in the absence of N_2_H_4_ under both light and dark conditions. No gas evolution was detected in either case (Figure S11, Supporting Information), confirming that the catalyst does not decompose by itself. Moreover, gas chromatography (GC) analysis (Figure 4d) of gases from the decomposition of N_2_H_4_·H_2_O catalyzed by Ni/NH_2_‐MIL‐125 detected only N_2_ and H_2_. To further determine N_2_H_4_·H_2_O conversion and H_2_ selectivity, UV–vis spectroscopy was used to quantify residual N_2_H_4_·H_2_O and detect possible NH_3_ formation. The results show that after the catalytic reaction, the amounts of N_2_H_4_·H_2_O and NH_3_ were determined to be 2.8 µmol and 1.05 µmol, respectively, corresponding to an N_2_H_4_·H_2_O conversion of over 99.86% and an H_2_ selectivity of 99.96% (Figure S12, Supporting Information). These results demonstrate that the catalyst achieves nearly complete N_2_H_4_·H_2_O conversion with almost 100% H_2_ selectivity.

To elucidate the pivotal role of visible light in promoting catalytic activity, the activity of Ni/NH_2_‐MIL‐125 was further evaluated under both illuminated and dark conditions (Figure 4e). Notably, even in the absence of light, the catalyst exhibited considerable performance, achieving complete dehydrogenation with a respectable TOF of 108.1 h^−1^ at 323 K. This result highlights the intrinsic catalytic activity of Ni/NH_2_‐MIL‐125. Subsequently, under visible light irradiation, the TOF more than doubles to 220.2 h^−1^, clearly demonstrating the positive effect of photoactivation. Importantly, temperature monitoring confirmed that the reaction system remained at constant 323 K throughout the visible light irradiation process (Figure S13, Supporting Information), effectively excluding photothermal effects as the origin of the enhanced activity. These findings unambiguously demonstrate that the enhancement in catalytic activity under visible light arises from an intrinsic photocatalytic process rather than photothermal effects. Specifically, photoexcitation of the NH_2_‐MIL‐125 support leads to the generation of electrons, which are subsequently transferred to Ni active sites. This electron transfer promotes proton reduction and accelerates the overall dehydrogenation process. For comparison, classic semiconductor‐supported Ni catalysts were also examined (Figure 4f). Ni/g‐C_3_N_4_ exhibited poor hydrogen selectivity and activity under visible light irradiation at 323 K, releasing only 1.39 equivalents of gas (H_2_ + N_2_) per mole of N_2_H_4_ over 72.9 min (TOF: 6.6 h^−1^). Ni/TiO_2_ showed better activity, achieving complete dehydrogenation within 9.98 min (TOF:120.2 h^−1^). Remarkably, Ni/NH_2_‐MIL‐125 shows the highest catalytic efficiency (220.2 h^−1^) among all tested samples, further highlighting the exceptional synergy between Ni NPs and the NH_2_‐MIL‐125 support under photocatalytic conditions. Additionally, systematic optimization revealed that a NaOH concentration of 2.5 M (Figure S14, Supporting Information) afforded the best catalytic performance under the tested conditions.

To further elucidate the roles of photogenerated electrons and holes in the catalytic process, charge quenching experiments were conducted under both visible light irradiation and dark conditions (Figure 4g). Silver nitrate (AgNO_3_) and potassium iodide (KI) were employed as specific scavengers for photogenerated electrons and holes, respectively.^[^ ^58^ ^]^ Under dark conditions, the addition of either scavenger had negligible effects on the activity of N_2_H_4_ dehydrogenation. In contrast, under visible light irradiation, the presence of AgNO_3_ drastically decreased the TOF from 220.2 to 109.0 h^−1^, nearly matching the dark value (108.1 h^−1^), indicating that the light‐induced activity enhancement is predominantly driven by photogenerated electrons. By comparison, the addition of KI resulted in only a slight decrease in activity, as I^−^ ions are oxidized by photogenerated holes to form I_2_/I_3_^−^ species, which mildly poison the Ni active sites. In addition, we carried out a more comprehensive ESR analysis to identify the possible radical species involved in the reaction. The ESR signals of superoxide radicals (•O_2_^−^) and hydroxyl radicals (•OH) were measured for both NH_2_‐MIL‐125 and Ni/NH_2_‐MIL‐125 under dark and visible‐light irradiation (Figure S15, Supporting Information). For •O_2_^−^, the signal intensity increased under light irradiation for both samples; However, Ni/NH_2_‐MIL‐125 exhibited a weaker signal than NH_2_‐MIL‐125. Meanwhile, •OH signals were negligible in all cases. These results indicate that •O_2_^−^ and •OH are not the dominant active species in this catalytic system. Instead, the enhanced catalytic activity under visible‐light irradiation more likely originates from direct electron transfer from the photoexcited NH_2_‐MIL‐125 to Ni, which increases the electron density at Ni sites and thereby facilitates N─H bond cleavage, rather than from radical‐driven pathways. Based on these findings, a plausible mechanism for N_2_H_4_ dehydrogenation over Ni/NH_2_‐MIL‐125 was proposed (Figure 4h). Upon visible light excitation, electrons in the valence band (VB) of NH_2_‐MIL‐125 are promoted to the conduction band (CB), simultaneously generating holes in the VB. Driven by the Schottky barrier formed at the NH_2_‐MIL‐125/Ni interface, photogenerated electrons migrate from the CB of NH_2_‐MIL‐125 to the Ni NPs, where they accumulate on the surface via electronic metal‐support interactions. Adsorbed N_2_H_4_ molecules then interact with these electron‐rich Ni sites, lowering the energy barrier for N─H bond cleavage (vide infra). Continuous generation of electron‐hole pairs under visible‐light irradiation sustains the catalytic reaction, promoting further N_2_H_4_ dehydrogenation.

**Figure 5:**
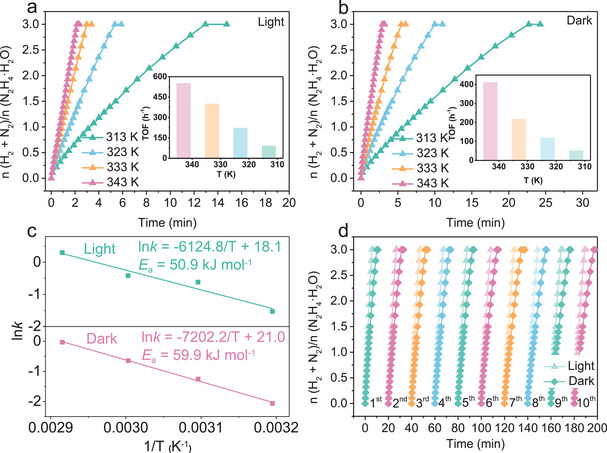
Time course plots and the corresponding TOF values for hydrogen evolution from aqueous N_2_H_4_ solution (0.4 m, 5 mL) catalyzed by Ni/NH_2_‐MIL‐125 with NaOH (3.0 m) at various temperatures a) in the dark and b) under visible light irradiation; c) The related Arrhenius plot versus 1/T; (d) Durability test over Ni/NH_2_‐MIL‐125 under light and dark at 323 K (*n*Ni/N_2_H_4_·H_2_O = 0.1).

The effect of temperature on the dehydrogenation rate of N_2_H_4_ catalyzed by Ni/NH_2_‐MIL‐125 was systematically investigated under both visible light irradiation (**Figure** **5**a) and dark conditions (Figure 5b) in the range of 313–343 K. In both cases, the reaction rate increased significantly with rising temperature. Arrhenius plots of ln*k* versus 1/T (Figure 5c) revealed an apparent activation energy (*E*_a_) of 50.9 kJ mol^−1^ under visible light irradiation, which is significantly lower than that determined in the dark (59.9 kJ mol^−1^), demonstrating that visible light irradiation effectively reduces the energy barrier for the N_2_H_4_ dehydrogenation. The enhanced activity can be attributed to the formation of electron‐rich Ni active sites induced by photogenerated electrons, which facilitate the activation and cleavage of N─H bonds in N_2_H_4_.

The cycling stability of Ni/NH_2_‐MIL‐125 was further evaluated to assess its practical applicability. After each run, an equal amount of N_2_H_4_·H_2_O substrate (100 µL) was replenished, and the tests were repeated under both light and dark conditions. As illustrated in Figure 5d and Figure S16 (Supporting Information), the catalyst maintained high catalytic activity with only a slight and gradual decrease over 10 consecutive cycles, and the hydrogen selectivity remained unchanged under both conditions, demonstrating its excellent cycling stability. Notably, in each cycle, the activity under visible light remained significantly higher than that in the dark, confirming the persistent promotional effect of photoactivation. Furthermore, after cycling, the catalyst was characterized by mass measurement, XRD, SEM, TEM, DLS, XPS, and ICP analyses. The average mass decreased from 77 to 60 mg after cycling (Figure S17, Supporting Information). Additionally, the XRD pattern (Figure S18a, Supporting Information) of the used catalyst shows that the characteristic diffraction peaks of the MOF structure nearly disappeared. Correspondingly, the SEM image (Figure S18b, Supporting Information) reveals that the catalyst partially transformed into fragmented structures, likely due to prolonged exposure to the strongly alkaline NaOH solution. TEM image (Figure S18c, Supporting Information) further corroborated these morphological changes, and the average diameter of Ni NPs shows a slight increase from 2.7 to 3.3 nm (Figure S18d, Supporting Information).

However, the particle size of the catalyst monitored by dynamic light scattering (DLS) at different reaction times shows no significant change (Figure S19, Supporting Information). Moreover, the Ni 2p and Ti 2p spectra (Figure S20, Supporting Information) indicate that the dominant chemical states of Ni and Ti are largely preserved, with the Ti^3+^/Ti^4+^ ratio changing only slightly (from 21.7% to 23.4%). The O 1s spectrum still shows the presence of oxygen vacancies with only minor variation in relative intensity (from 28.9% to 28.7%). ICP‐MS results (Table S2, Supporting Information) show that the Ti and Ni contents of the catalyst show no significant changes before (29.1 wt.% Ti and 24.7 wt.% Ni) and after cycling test (28.9 and 25.2 wt.%), respectively. Overall, although some structural degradation occurs during cycling, the particle size distribution, the key catalyst composition, metal contents, and valence states remain essentially unchanged, consistent with its good catalytic recycling performance.

**Figure 6:**
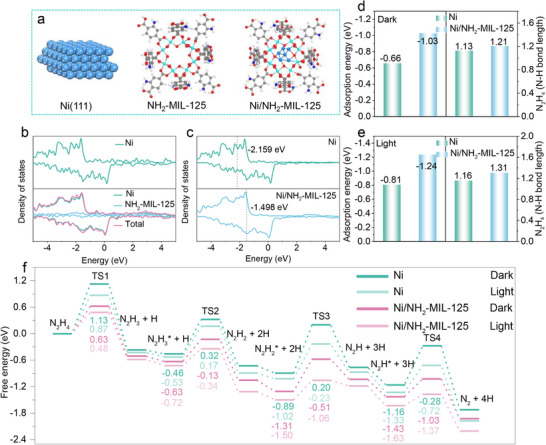
a) The optimized structural models of Ni(111), NH_2_‐MIL‐125, and Ni/NH_2_‐MIL‐125; b,c) The DOS of Ni and Ni/NH_2_‐MIL‐125 with the dashed line representing the ɛd; The adsorption energy and N‐H bond length of N_2_H_4_ for Ni NPs and Ni/NH_2_‐MIL‐125 d) in the dark and e) under visible light irradiation; f) Energy profiles of N_2_H_4_ molecule dissociation on the Ni NPs and Ni/NH_2_‐MIL‐125 surfaces in the dark and under visible light irradiation.

To gain further insight into the mechanism of visible‐light‐promoted N_2_H_4_ dehydrogenation over Ni/NH_2_‐MIL‐125, DFT calculations were performed. In the light‐excitation model, two additional electrons were introduced into the system to simulate the excited‐state electronic structure under illumination relative to that in the dark. **Figure** **6**a presents the optimized structures of Ni NPs, NH_2_‐MIL‐125, and the Ni/NH_2_‐MIL‐125 samples. Density of states (DOS) analysis (Figure 6b,c) reveals that near the Fermi level, the DOS of Ni/NH_2_‐MIL‐125 is dominated by contributions from Ni active sites rather than the NH_2_‐MIL‐125 framework, indicating that the adsorption of reactants and intermediates occurs primarily on Ni active sites, confirming their role as active centers. Importantly, interfacial electronic interactions between Ni and NH_2_‐MIL‐125 significantly modulate the d‐band center (εd), shifting it from −2.159 eV (for Ni NPs) to −1.498 eV (for Ni/NH_2_‐MIL‐125). The upshift strengthens the chemisorption of the reactants/intermediates at the active sites.^[^ ^59^, ^60^ ^]^ Furthermore, adsorption energy calculations further support this conclusion. Under dark conditions (Figure 6d), N_2_H_4_ adsorbs more strongly on Ni/NH_2_‐MIL‐125 (−1.03 eV) than on Ni NPs (−0.66 eV), accompanied by a longer N─H bond (1.21 vs 1.13 Å), suggesting enhanced activation due to the strong interaction between Ni and NH_2_‐MIL‐125. Upon visible‐light irradiation (Figure 6e), adsorption becomes even stronger (−1.24 eV for Ni/NH_2_‐MIL‐125; ‐0.81 eV for Ni) and N─H bonds are further elongated (1.31 and 1.16 Å), respectively. These results indicate that visible light further optimizes the electronic structure of catalysts, thereby enhancing the adsorption and activation of N_2_H_4_.

Subsequently, the adsorption and dissociation processes of N_2_H_4_ over Ni NPs and Ni/NH_2_‐MIL‐125 were systematically investigated under both dark and light irradiation conditions. As illustrated in Figure 6f; Figures S21 and S22 (Supporting Information), the N_2_H_4_ decomposition process on both catalysts proceeds via a stepwise intramolecular dehydrogenation pathway: N_2_H_4_ → N_2_H_3_ + H, N_2_H_3_ → N_2_H_2_ + H, N_2_H_2_→ N_2_H + H, N_2_H → N_2_ + H. Notably, the catalytic decomposition of N_2_H_2_ was found to be exothermic under both dark and light irradiation conditions. Under dark conditions (Figure 6f), unsupported Ni NPs show relatively high energy barriers for the dehydrogenation steps (1.13 eV for N_2_H_4_, 0.78 eV for N_2_H_3_, 1.09 eV for N_2_H_2_, and 0.88 eV for N_2_H). Among these steps, the cleavage of N─H bonds in N_2_H_4_ and N_2_H_2_ is energetically demanding, representing the rate‐determining steps in the overall reaction, consistent with previous reports.^[^ ^61^ ^]^ In contrast, when Ni NPs are immobilized on NH_2_‐MIL‐125, the energy barriers are substantially reduced to 0.63 eV for N_2_H_4_, 0.50 eV for N_2_H_3_, 0.80 eV for N_2_H_2_, and 0.40 eV for N_2_H, respectively, demonstrating that the introduction of support can enhance N─H bond activation even in the absence of light. Visible‐light irradiation further reduces the energy barriers for both catalysts, suggesting a photo‐induced promotional effect. Most notably, the Ni/NH_2_‐MIL‐125 catalyst under visible‐light irradiation exhibits the lowest energy barriers across all steps: 0.48 eV for N_2_H_4_, 0.38 eV for N_2_H_3_, 0.44 eV for N_2_H_2_, and 0.26 eV for N_2_H. Overall, the enhanced catalytic activity of Ni/NH_2_‐MIL‐125 under visible light irradiation is attributed to a synergistic effect: the NH_2_‐MIL‐125 support modulates the electronic structure of Ni sites, enhancing the activation of intermediates; simultaneously, the light‐induced generation of electron‐rich active centers further lowers the reaction energy barriers, collaboratively promoting N_2_H_4_ dehydrogenation.

## 3 Conclusion

In summary, we have successfully developed a non‐precious metal catalyst for visible‐light‐promoted N_2_H_4_·H_2_O dehydrogenation by immobilizing Ni NPs onto a visible‐light‐responsive NH_2_‐MIL‐125 framework. The resulting Ni/NH_2_‐MIL‐125 catalyst exhibits an outstanding performance under light irradiation, achieving complete N_2_H_4_ dehydrogenation with a high TOF of 220.2 h^−1^ at 323 K, which is more than twice the activity observed in the dark (108.1 h^−1^) and superior to most reported non‐precious metal catalysts. Combined results from experimental characterization and DFT calculations reveal that the exceptional catalytic performance arises from the effective dispersion of Ni NPs and the light‐induced electron transfer from the MOF to the Ni active sites, which synergistically enhance N_2_H_4_ adsorption and lower the energy barrier for N─H bond cleavage. This work offers a promising strategy for the design of cost‐effective, efficient, and stable catalysts for N_2_H_4_·H_2_O dehydrogenation, advancing its practical application as a viable hydrogen storage material.

## 4 Experimental Section

### Synthesis of Ni/NH_2_‐MIL‐125

NH_2_‐MIL‐125 was prepared by a solvothermal method (details provided in the Supporting Information). Ni/NH_2_‐MIL‐125 was synthesized via a simple wet‐chemical reduction approach. In a typical synthesis, NH_2_‐MIL‐125 (72 mg) was dispersed in deionized water (5 mL), followed by the addition of NiCl_2_·6H_2_O (48.4 mg, 0.2 mmol). The suspension was ultrasonicated for 30 min at ambient temperature to ensure homogeneous dispersion. Subsequently, NaBH_4_ (60 mg) was added under vigorous stirring. The reduction was allowed to proceed until gas evolution ceased, affording the Ni/NH_2_‐MIL‐125 catalyst.

For the synthesis of control catalysts, unsupported Ni NPs were prepared following the same procedure as described above, but without any support. Ni NPs immobilized on other photoactive supports, including TiO_2_ and g‐C_3_N_4_, were prepared by replacing NH_2_‐MIL‐125 with the respective supports under otherwise identical conditions. A physical mixture of Ni and NH_2_‐MIL‐125 (Ni + NH_2_‐MIL‐125) was also prepared by directly mixing the preformed Ni NPs with NH_2_‐MIL‐125.

### Catalytic Activity Measurement

As shown in Figure S23, catalytic dehydrogenation of N_2_H_4_·H_2_O was conducted in a quartz reactor containing Ni/NH_2_‐MIL‐125 (0.2 mmol Ni) and NaOH solution (2.5 M, 5 mL). A 300 W Xe lamp equipped with a 420 nm cut‐off filter (CEL‐HXF300, Beijing Zhongjiao Jinyuan Technology Co., Ltd.) was positioned on the top of the reactor as the visible‐light source, while the reaction temperature was maintained at 323 K by a circulating water system. Upon addition of N_2_H_4_·H_2_O, the reaction was initiated, and the evolved H_2_ was quantified by the water displacement method. Before measurement, the gas was passed through a trap containing 1.0 M HCl solution to remove any possible ammonia. The molar amount of Ni in all catalytic reactions was fixed at 0.2 mmol. The H_2_ selectivity (α) was calculated according to the following equations (Equations (3) and (4)):

(3) $$N_{2}H_{4}\to 2\alpha H_{2}+\left(2\alpha +1\right)/\;3N_{2}+4\left(1-\alpha \right)/\;3NH_{3}$$

(4) $$\alpha =\frac{3x-1}{8}\left[x=\frac{n\left(H_{2}+N_{2}\right)}{n\left(N_{2}H_{4}\right)}\left(\frac{1}{3}\leq \;x\;\leq \;3\right)\right]$$

For recycling tests, an equal amount of N_2_H_4_·H_2_O (2.0 mmol) was directly injected into the reaction flask after completion of each cycle, and the procedure was repeated for ten cycles at 323 K. After the last run, the catalyst was collected by centrifugation, washed with water, dried under vacuum, and examined by XRD and TEM.

## Conflict of Interest

The authors declare no conflict of interest.

## Supporting information



Supporting Information

## Data Availability

The data that support the findings of this study are available from the corresponding author upon reasonable request.
