# Supplementary material for: Light‐Promoted Hydrazine Dehydrogenation over Ni/NH2‐MIL‐125: Unraveling Mechanisms for Efficient Hydrogen Production
Source: Adv Sci (Weinh). 2025 Dec 5;13(11):e19320. doi: 10.1002/advs.202519320 (PMC12931232; doi:10.1002/advs.202519320)
Supplement: Supplementary file 1 — Supporting Information [file ADVS-13-e19320-s001.docx]

**Supporting Information**

**Light-promoted Hydrazine Dehydrogenation over Ni/NH_2_-MIL-125: Unraveling Mechanisms for Efficient Hydrogen Production**

*Jianjun Long^a^, Caili Hu^a^, Qilu Yao*^,a^, Kun Wang^a^, Gang Feng^b^, Shaowen Cao^c^, Zhang-Hui Lu*^,a^*

*^a^*Key Laboratory of Green Catalysis of Jiangxi Education Institutes, Key Laboratory of Green Hydrogen and Advanced Catalysis of Jiangxi Province, Key Laboratory of Fluorine and Silicon for Energy Materials and Chemistry of Ministry of Education College of Chemistry and Materials, Jiangxi Normal University, Nanchang 330022, China

*^b^*Key Laboratory for Environment and Energy Catalysis of Jiangxi Province, College of Chemistry and Chemical Engineering, Nanchang University, Nanchang 330031, China

*^c^*State Key Laboratory of Advanced Technology for Materials Synthesis and Processing, Wuhan University of Technology, Wuhan 430070, China

Emails: yaoqilu@jxnu.edu.cn (Q. Yao); luzh@jxnu.edu.cn (Z.-H. Lu)

**Table of Contents**

**Experimental Details**

**Supporting Figures**

Figure S1. EDX pattern of Ni/NH_2_-MIL-125.

Figure S2. TEM images and particle size distribution of Ni NPs.

Figure S3. XRD patterns and EPR spectra of Ni/NH_2_-MIL-125-*x* (*x* = 0-80 mg).

Figure S4. Partial enlarged images of BET for NH_2_-MIL-125 and Ni/NH_2_-MIL-125.

Figure S5. Ni 2p spectra of Ni/NH_2_-MIL-125 after Ar^+^ etching for different time.

Figure S6. O 1s spectra of NH_2_-MIL-125 and Ni/NH_2_-MIL-125.

Figure S7. UV-Vis diffuse reflectance spectroscopy, PL spectra, EIS, and Transient photocurrent response of MIL-125, and Ni/MIL-125.

Figure S8. Catalytic activity of N_2_H_4_ dehydrogenation for commercial Ni powder.

Figure S9. Catalytic activity of N_2_H_4_ dehydrogenation for NiO.

Figure S10. Catalytic activity of N_2_H_4_ with Ni/NH_2_-MIL-125 and Ni/MIL-125.

Figure S11. Gas evolution from Ni/NH_2_-MIL-125 without N_2_H_4_ under light and dark.

Figure S12. UV-vis absorption spectra and standard curves of N_2_H_4_ and NH_4_^+^.

Figure S13. Temperature of the photocatalytic system before and after the reaction.

Figure S14. Catalytic activity of N_2_H_4_ with different concentration of NaOH.

Figure S15. ESR spectra of NH_2_-MIL-125 and Ni/NH_2_-MIL-125 under light and dark.

Figure S16. Durability test over Ni/NH_2_-MIL-125 under light and dark at 323 K.

Figure S17. The mass of the Ni/NH_2_-MIL-125 before and after the durability test.

Figure S18. XRD, SEM, and TEM images of catalyst after the durability test.

Figure S19. DLS of Ni/NH_2_-MIL-125 in the hydrazine dehydrogenation medium.

Figure S20. Ni 2p, Ti 2p, N 1s, and O 1s of Ni/NH_2_-MIL-125 after the durability test.

Figure S21. Calculated intermediate structures for the reactions of N_2_H_4_ with Ni.

Figure S22. Calculated intermediate structures for the reactions of N_2_H_4_ with Ni/NH_2_-MIL-125.

Figure S23. Diagram of catalytic dehydrogenation device.

**Supporting Table**

Table S1. Comparison of the catalytic activities for N_2_H_4_ dehydrogenation of in aqueous solution with previously reported non-precious catalysts.

Table S2. ICP-MS analysis of Ni/NH_2_-MIL-125 before and after durability testing in N_2_H_4_ dehydrogenation.

**Supporting References**

**Experimental Section**

**Chemicals**

All the chemicals were analytical grade and used as received without further purifications. Nickel (II) chloride hexahydrate (NiCl_2_·6H_2_O, 99.9%, Aladdin), titanium butoxide (C_16_H_36_O_4_Ti, ≥99.0%, Aladdin), 2-amino-1,4-benzenedicarboxylic acid (C_8_H_7_NO_4_, 98%, Aladdin), terephthalic acid (C_8_H_6_O_4_, 99%, Aladdin), melamine (C_3_H_6_N_6_, 99%, Aladdin), hydrazine monohydrate (N_2_H_4_·H_2_O, 98%, Aladdin), sodium borohydride (NaBH_4_, 98%, Aladdin), sodium hydroxide (NaOH, ≥96.%, Aladdin), commercial Ni powder (325 mesh, 99.8%, Innochem), p-dimethylaminobenzaldehyde ((CH_3_)_2_NC_6_H_4_CHO, AR, Aladdin), salicylic acid (C_7_H_6_O_3_, 99.5%, Aladdin), sodium citrate dihydrate (C_6_H_5_Na_3_O_7_·2H_2_O, 99.0%, Aladdin), sodium hypochlorite solution (0.05 M, Pinggen Technology Reagent Co., Ltd.), methanol (CH_3_OH, ≥99.5%, Tianjin ZhiYuan Reagent Co., Ltd), and N,N-Dimethylformamide (DMF, ≥99.5%, Tianjin ZhiYuan Reagent Co., Ltd) were used as received. Ultrapure water with a resistivity of 18.0 MΩ·cm was obtained by reversed osmosis followed by ion exchanged and filtration.

**Synthesis of NH_2_-MIL-125**

NH_2_-MIL-125 was synthesized following a solvothermal procedure. Specifically, 2-amino-1,4-benzenedicarboxylic acid (NH_2_-BDC, 1.05 g) was dissolved in a 3:7 (v/v) mixed solvent (50.00 mL) of methanol and N, N-dimethylformamide (DMF). Titanium butoxide (TBOT, 1.25 mL) was then added to the homogeneous solution under continuous sonication for 60 min at ambient temperature. The resulting mixture was transferred into in a 100 mL Teflon-lined stainless-steel autoclave and heated at 120 °C for 12 h. The yellow precipitated was collected by centrifugation (8,000 rpm, 5 min) and washed alternately with anhydrous DMF and methanol three times, and subsequently dried under vacuum at 40 ℃ for 12 h to remove residual solvents.

**Syntheses of MIL-125**

A mixture of 6 mL of methanol and 54 mL of DMF were added to a 250 mL round bottom flask and stirred uniformly. Then, 3.0 g of terephthalic acid was dissolved in the above mixed solution. After ultrasound for 10 min, 1.56 mL of titanium butoxide was added as a titanium source, and then stirring for 10 min until the solution is clear and transparent. The uniformly mixed solution was transferred to a 200 ml Teflon-liner and placed into a temperature-controlled oven and heated at 150 °C for 24 h. After the hydrothermal reaction, reaction system was cooled to room temperature, and the MIL-125 in suspension was obtained by centrifuging and washed with DMF and methanol several times. The final samples were placed in a vacuum drying oven at 40 °C for 12 hours.

**Synthesis of TiO_2_**

TiO_2_ nanosheets were prepared by a simple hydrothermal method. Typically, 10 mL of titanium butoxide was mixed with 1.6 mL HF with stirring for 30 min. Then the solution was transferred into a 50 mL Teflon-lined autoclave and reaction at 180 °C for 24 h. After that, the resulting white slurry was centrifuged and washed with distilled water several times and dried in a vacuum oven.

**Synthesis of g-C_3_N_4_**

5.0 g of melamine was placed in a porcelain boat and heated to 550 ℃ at a rate of 5 °C/min in muffle furnace. The temperature was maintained at 550 ℃ for 10 h. After cooling to room temperature, the resulting yellow bulk solid was ground into a fine powder using a mortar.

**Synthesis of NiO NPs and NiO + NH_2_-MIL-125**

NiO NPs were synthesized by oxidizing the Ni NPs obtained from the NaBH_4_ reduction of NiCl_2_·6H_2_O, followed by calcination at 500 °C for 4 h in a muffle furnace. The resulting NiO NPs were then mixed with NH_2_-MIL-125 and subjected to ultrasonication for 30 min to obtain the NiO + NH_2_-MIL-125 composite.

**Characterization**

The morphology of samples were investigated by scanning electron microscopy (SEM, SU-8020). Transmission electron microscopy (TEM) combined with energy dispersive X-ray (EDX) detector and mapping analyses were recorded on JEM-2100 with Super-X EDS system under operating voltages of 300 kV. Powder X-ray diffraction (XRD) measurements were performed on a Rigaku Rint 2200 X-ray diffractometer with Cu Kα source (40 KV, 200mA). Fourier transform infrared (FTIR) spectra were carried on a Thermo Nicolet 6700 spectrometer. Electron paramagnetic resonance (EPR) was carried out by using Bruker A300 at 77 K,and electron spin resonance (ESR) was carried out by using BrukerEMXplus-6/1. Surface area and pore size distribution were measured by N_2_ adsorption-desorption at 77 K using a micromeritics Autosorb iQ analyzer. X-ray photoelectron spectroscopy (XPS) was performed on an ESCALAB 250Xi spectrometer with an Al Kα X-ray source after Ar sputtering. Gas compositions were determined by using a gas chromatograph (GC-9790Ⅱ) with a thermal conductivity detector (TCD) and a TDX-01 chromatographic column (oven temperature: 333 K, detector temperature 393K). The dynamic light scattering of Ni/NH_2_-MIL-125 in the hydrazine dehydrogenation medium was measured by Zetasizer Lab (Malvern Panalytical Ltd). The contents of Ni and Ti in catalysts were determined using Agilent 7850 inductively coupled plasma-mass spectrometry (ICP-MS). Photoelectrochemical measurements were performed on an electrochemical workstation (CHI 760E, CH Instruments) with the 300 W Xenon lamp (CEL-HXF300, Beijing Zhongjiao Jinyuan Technology Co., Ltd.) as the light source at room temperature. A conventional standard three-electrode system was used. For testing, 5 mg of sample was dispersed in 3 mL of ethanol solution and sonicated for 1 h. Drops of the solution were applied to an ITO glass with an area of 1 cm^2^. The sample-coated ITO was used as the working electrode, a platinum sheet (1*1 cm^2^) was used as the counter electrode, and a saturated calomel electrode (SCE) was used as the reference electrode. The electrolyte was 0.5 M aqueous sodium sulfate. Electrochemical impedance spectroscopy (EIS) tests were performed in the frequency range of 105 to 10^-2^ Hz with an amplitude of 5 mV. Photocurrent measurements were performed at a rate of 0.1 A s^-1^ over a period of 0 to 520 seconds.

**Calculation method of TOF**

The turn over frequency (TOF) reported in this work is an apparent TOF value based on the number of metal (Ni) atoms in catalysts, which is calculated from the equation as follows:

$$\text{TOF= }\frac{\text{n }\left( \text{H}_{\text{2}} \right)}{\text{n }\left( \text{metal} \right)\text{×t}}\text{ (S1)}$$

Where *n*H_2_ is the mole number of generated H_2_, *n*metal is the total mole number of Ni in catalyst and t is the completed reaction time in hour.

**Density Functional Theory (DFT) Calculations**

The Vienna Ab Initio Package (VASP) was employed to perform the density functional theory (DFT) calculations within the generalized gradient approximation (GGA) using the Perdew, Burke, and Enzerhof (PBE) formulation.^[S1-S3]^ The projected augmented wave (PAW) potentials were applied to describe the ionic cores and take valence electrons into account using a plane wave basis set with a kinetic energy cutoff of 520 eV.^[S4,S5]^ Partial occupancies of the Kohn–Sham orbitals were allowed using the Gaussian smearing method with a width of 0.05 eV. The electronic energy was considered self-consistent when the energy change was smaller than 10^−5^ eV. A geometry optimization was considered convergent when the force change was smaller than 0.05 eV/Å. In our structure, the U correction is used for Ni (4.91 eV) atoms. Grimme’s DFT-D3 methodology was used to describe the dispersion interactions.^[S6]^ The vacuum spacing perpendicular to the plane of the structure is 30 Å. The Brillouin zone integral utilized the surfaces structures of 2×2×1 monkhorst pack K-point sampling. Finally, the adsorption energies (Eads) were calculated as Eads = Ead/sub -Ead -Esub, where Ead/sub, Ead, and Esub are the total energies of the optimized adsorbate/substrate system, the adsorbate in the structure, and the clean substrate, respectively. The free energy was calculated using the equation:

G=Eads+ZPE-TS

where G, Eads, ZPE and TS are the free energy, total energy from DFT calculations, zero point energy and entropic contributions, respectively. For excited state structures, full optimization is performed by applying two electrons to the system.

**Supporting Figures**

**Figure S1.** EDX pattern of Ni/NH_2_-MIL-125.

**Figure S2.** (a-c) TEM images and (d) particle size distribution of Ni NPs.

**Figure S3**. (a) XRD patterns and (b) EPR spectra of Ni/NH_2_-MIL-125 prepared with different amounts of NaBH_4_.

**Figure S4**. Partial enlarged images of N_2_ adsorption/desorption for NH_2_-MIL-125 and Ni/NH_2_-MIL-125.

**Figure S5**. Ni 2p spectra of Ni/NH_2_-MIL-125 after Ar^+^ etching for different times.

**Figure S6**. (a) O 1s spectra of NH_2_-MIL-125 and Ni/NH_2_-MIL-125.

**Figure S7**. (a) UV-Vis diffuse reflectance spectroscopy, (b) PL spectra, (c) EIS, and (d) Transient photocurrent response of MIL-125, NH_2_-MIL-125, Ni/MIL-125, and Ni/NH_2_-MIL-125.

**Figure S8**. (a) Time course plots for hydrogen evolution from aqueous N_2_H_4_ solution (0.4 M, 5 mL) over commercial Ni powder under light in the presence of NaOH (2.5 M) at 323 K (*n*Ni/*n*N_2_H_4_ = 0.1); (b) The XRD pattern of commercial Ni powder.

As shown in Figure S8a, commercial Ni powder exhibits no dehydrogenation activity, which can be attributed to its high crystallinity (Figure S8b) and large particle size (325 mesh, ~44 μm).

**Figure S9**. (a) Time course plots for hydrogen evolution from aqueous N_2_H_4_ solution (0.4 M, 5 mL) over NiO and NiO + NH_2_-MIL-125 under light in the presence of NaOH (2.5 M) at 323 K (*n*Ni/*n*N_2_H_4_ = 0.1); (b) The XRD pattern of NiO.

To further determine whether NiO participates in the catalytic reaction, Ni NPs were calcined to obtain NiO and their catalytic performance was evaluated. The results show that NiO exhibits almost no catalytic activity (Figure S9), and even when supported on the NH_2_-MIL-125, it remains inactive. These findings indicate that NiO is not the actual active species in the reaction.

**Figure S10.** Time course plots for hydrogen evolution from aqueous N_2_H_4_ solution (0.4 M, 5 mL) over Ni/NH_2_-MIL-125 and Ni/MIL-125 under light in the presence of NaOH (2.5 M) at 323 K (*n*Ni/*n*N_2_H_4_ = 0.1).

**Figure S11.** Time course plots for hydrogen evolution from Ni/NH_2_-MIL-125 under (a) light and (b) dark in the presence of NaOH (2.5 M) at 323 K (*n*Ni/*n*N_2_H_4_ = 0.1).

**Figure S12.** UV-vis absorption spectra and corresponding standard curves of (a,b) N_2_H_4_·H_2_O and (c,d) NH_4_^+^ at different concentrations.

**Determination of** **N_2_H_4_·H_2_O conversion:** A colorimetric reagent for N_2_H_4_·H_2_O was prepared by dissolving 0.5 g of p-dimethylaminobenzaldehyde in a mixture of 25 mL of anhydrous ethanol and 2.5 mL of HCl (12 M). A series of standard N_2_H_4_·H_2_O solutions (20, 16, 12, 8, 6, 4, and 2 μmol·mL^-1^) were prepared by diluting appropriate volumes of N_2_H_4_·H_2_O (100, 80, 60, 40, 30, 20, and 10 μL) to 100 mL with deionized water. For the color development, 34 mL deionized water, 100 μL of the standard or reaction solution, and 2 mL of the colorimetric reagent were mixed and left to stand for 1 h. The UV-Vis absorption spectra were recorded, and a standard calibration curve was established. Based on the standard curve of N_2_H_4_·H_2_O and the analysis of the reaction solution, the concentration of residual N_2_H_4_·H_2_O was determined to be 0.56 μmol mL^-1^. Given a total reaction volume of 5 mL, the total residual amount was calculated to be 2.8 μmol (0.56 μmol mL^-1^ × 5). Since the initial amount of N_2_H_4_·H_2_O added was 2.0 mmol (2000 μmol), the conversion was calculated to be 99.86% ((2000-2.8)/2000× 100%), indicating a nearly complete conversion of N_2_H_4_·H_2_O.

**Determination of H_2_ Selectivity:** H_2_ selectivity was evaluated by monitoring the formation of possible NH_3_ during the reaction. The amount of NH_3_ was determined by measuring the concentration of NH_4_^+^ ions in the reaction solution. Specifically, a colorimetric method was employed using the following reagent solutions: (A) 1 M sodium hydroxide solution containing 5 wt% salicylic acid and 5 wt% sodium citrate dihydrate, (B) 0.05 M sodium hypochlorite solution, and (C) 1 wt% sodium nitroferricyanide solution. NH_4_Cl standard solutions with concentrations (0.04, 0.025, 0.02, 0.01, 0.005, and 0.0025 μmol mL^-1^) were prepared. For the assay, 2 mL of each standard solution (or the reaction solution) was transferred into a 10 mL centrifuge tube, followed by the sequential addition of 2 mL of reagent A, 1 mL of reagent B, and 0.2 mL of reagent C. After allowing the mixture to stand for 1 h, the UV-Vis absorption spectra were recorded, and a standard curve was established based on the absorbance values at different NH_4_^+^ concentrations. Based on the reaction solution measurements and the NH_4_^+^ standard curve, the concentration of NH_4_^+^ in the reaction solution was calculated to be 0.21 (0.021*10) μmol mL^-1^. Given the total reaction volume of 5 mL, the total amount of NH_3_ produced was determined to be 1.05 μmol (0.21 × 5). According to the reaction equation 3N_2_H_4_(l) → 4NH_3_(g) + N_2_(g), 3 mol of N_2_H_4_ produces 4 mol of NH_3_. Based on this stoichiometric ratio, the amount of N_2_H_4_·H_2_O converted to NH_3_ was calculated to be 0.7875 μmol. The total amount of N_2_H_4_·H_2_O added in each reaction was 2.0 mmol (2000 μmol). Therefore, the H_2_ selectivity for the decomposition of N_2_H_4_ to H_2_ and N_2_ was calculated to be 99.96% ((2000-0.7875)/2000 × 100%), indicating an almost 100% selectivity toward H_2_.


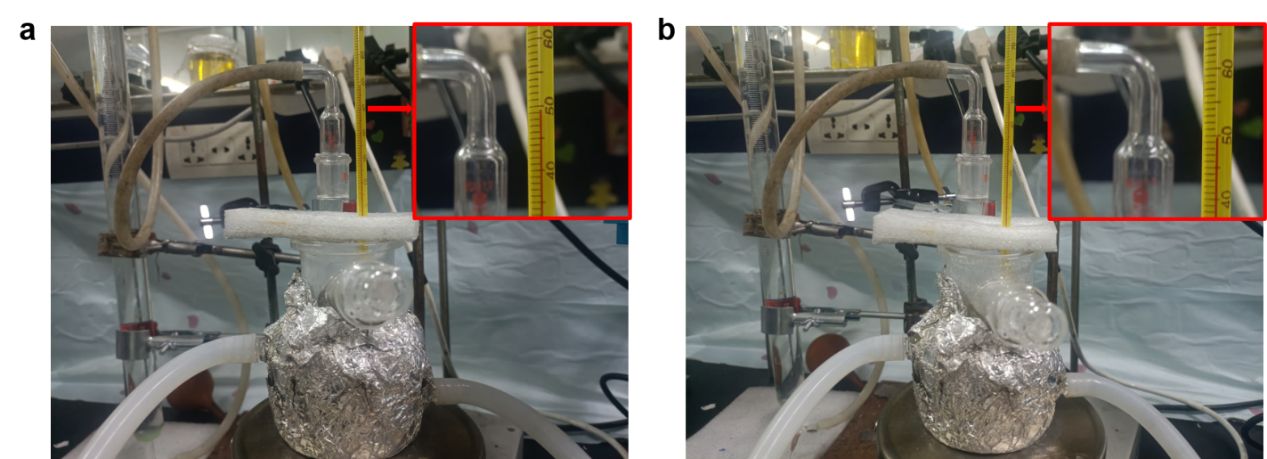


**Figure S13.** Temperature of the photocatalytic system (a) before and (b) after the reaction.

The temperature of the catalytic system before and after the catalytic reaction was tested using a thermometer. After removing the light source above, the thermometer was inserted into the reaction solution, and the temperature before the reaction was 49.5 ℃ (322.5 K), and the temperature after the reaction was 49.8 ℃ (322.8 K) , with almost no difference between the two.

**Figure S14.** (a)Time course plots for hydrogen evolution from aqueous N_2_H_4_ solution (0.4 M, 5 mL) over Ni/NH_2_-MIL-125 under light with different concentration of NaOH at 323 K and (b) the corresponding TOF values (*n*Ni/*n*N_2_H_4_ = 0.1).

NaOH in the catalytic process acts as a promoter for hydrogen production reaction. The possible reason is that the existence of NaOH can effectively reduce the concentration of undesired N_2_H_5_^+^ (N_2_H_5_^+^ + OH^-^ → N_2_H_4_ + H_2_O) and accelerate the rate-determining step (N_2_H_4_ → N_2_H_3_* + H*), thus promoting reaction kinetics. In addition, the presence of NaOH can also suppressed the release of by-product NH_3_ as well.

**Figure S15.** ESR spectra of NH_2_-MIL-125 and Ni/NH_2_-MIL-125 under light and dark.

**Figure S16.** Durability test over Ni/NH_2_-MIL-125 under (a,b) light and (c,d) dark at 323 K (*n*Ni/*n*N_2_H_4_ = 0.1).

**Figure S17.** The mass of the Ni/NH_2_-MIL-125 before and after the durability test.

**Figure S18.** (a) XRD patterns of Ni/NH_2_-MIL-125 before and after the durability test. (b) SEM image and (c,d) TEM images of Ni/NH_2_-MIL-125 after the durability test. The inset of (d) shows the particle size distribution.

**Figure S19.** Dynamic light scattering analysis of Ni/NH_2_-MIL-125 in the hydrazine dehydrogenation.

**Figure S20.** (a) Ni 2p, (b) Ti 2p, (c) N 1s, and (d) O 1s of Ni/NH_2_-MIL-125 after the durability test.

**Figure S21.** The calculated intermediate structures for the reactions of N_2_H_4_ with Ni(111).

**Figure S22.** The calculated intermediate structures for the reactions of N_2_H_4_ with Ni/NH_2_-MIL-125.

**Figure S23.** Diagram of catalytic dehydrogenation device.

**Supporting Table**

**Table S1.** Comparison of the catalytic activities for N_2_H_4_ dehydrogenation of in aqueous solution with previously reported non-precious catalysts.

| **Catalysts** | **T(K)** | **Selectivity (%)** | **TOF (h^-1^)** | ***E*_a_ (kJ mol^-1^)** | **Ref.** |
| --- | --- | --- | --- | --- | --- |
| Ni/Al_2_O_3_-HT | 303 | 93 | 2.2 | 47.0 | S7 |
| NiCo/NiO-CoO_x_ | 298 | 99 | 5.49 | 45.15 | S8 |
| 2D-NiFe/CeO_2_ | 323 | 99 | 5.73 | 44.06 | S9 |
| Ni Nanofibers | 333 | 100 | 6.9 | 52.07 | S10 |
| NiMoB-La(OH)_3_ | 323 | 100 | 13.3 | 55.1 | S11 |
| Cu@Ni_5_Fe_5_ | 343 | 100 | 18.2 | 79.2 | S12 |
| Ni-CNTs-OH | 333 | 100 | 19.4 | 51.05 | S13 |
| NiFeMo | 323 | 100 | 28.8 | 50.7 | S14 |
| Ni-W-O | 323 | 99 | 33 | / | S15 |
| Ni/CeO_2_ | 323 | 100 | 34 | 56.2 | S16 |
| NiFe/Cu | 343 | 100 | 35.3 | 44 | S17 |
| Ni_3_Cr/NiO-CrO_x_ | 323 | 100 | 39.8 | 53.1 | S18 |
| Ni-0.080CeO_2_ | 303 | 99 | 51.6 | 47.0 | S19 |
| Ni@TNTs | 333 | 100 | 96.0 | 53.2 | S20 |
| NiFe-La(OH)_3_ | 343 | 100 | 100.6 | 57.8 | S21 |
| CuNi/La_2_O_2_CO_3_/rGO | 343 | 100 | 114.3 | 65.54 | S22 |
| Ni-CeO_2_@SiO_2_ | 343 | 100 | 219.5 | 59.26 | S23 |
| **Ni/NH_2_-MIL-125** | **323** | **100** | **220.2** | **50.9** | **This work** |
| Ni/TiO_2_ | 343 | 100 | 265.49 | 58.67 | S24 |
| Ni-Cr(OH)_3_/C-TiO_2_ | 323 | 100 | 266 | 52.6 | S25 |

**Table S2.** ICP-MS analysis of Ni/NH_2_-MIL-125 before and after durability testing in N_2_H_4_ dehydrogenation.

| **Catalysts** | **Ti (wt%)** | **Ni (wt%)** |
| --- | --- | --- |
| Ni/NH_2_-MIL-125-1^st^ | 29.1 | 24.7 |
| Ni/NH_2_-MIL-125-10^th^ | 28.9 | 25.2 |

**References**

1. G. Kresse, J. Furthmüller, *Phys. Rev. B* **1996**, 54, 11169-11186.
2. J. P. Perdew, K. Burke, M. Ernzerhof, *Phys. Rev. Lett.* **1996**, 77, 3865-3868.
3. G. Kresse, D. Joubert, *Phys. Rev. B* **1999**, 59, 1758-1775.
4. P. E. Blöchl, *Phys. Rev. B* **1994**, 50, 17953-17979.
5. S. Grimme, J. Antony, S. Ehrlich, H. J. Krieg, *Chem. Phys.* **2010**, 132, 154104.
6. G. Henkelman, B. P. Uberuaga, H. J. Jonsson, *Chem. Phys.* **2000**, 113, 9901.
7. L. He, Y. Huang, A. Wang, X. Wang, X. Chen, J.J. Delgado, T. Zhang, *Angew. Chem. Int. Ed.* **2012**, 51, 6191-6194.
8. D. Wu, M. Wen, X. Lin, Q. Wu, C. Gu, H. Chen, *J. Mater. Chem. A* **2016**, 4, 6595-6602.
9. D. Wu, M. Wen, C. Gu, Q. Wu, *ACS Appl. Mater. Interfaces* **2017**, 9, 16103–16108.
10. Q. Fu, P. Yang, J. Wang, H. Wang, L. Yang, X. Zhao, *J. Mater. Chem. A* **2018**, 6, 11370-11376.
11. J. Zhang, Q. Kang, Z. Yang, H. Dai, D. Zhuang, P. Wang, *J. Mater. Chem. A* **2013**, 1, 11623-11628.
12. J. Wang, Y. Li, Y. Zhang, *Adv. Funct. Mater.* **2014**, 24, 7073-7077.
13. P. Yang, L. Yang, Q. Gao, Q. Luo, X. Zhao, X. Mai, Q. Fu, M. Dong, J. Wang, Y. Hao, R. Yang, X. Lai, S. Wu, Q. Shao, T. Ding, J. Lin, Z. Guo, *Chem. Commun.* **2019**, 55, 9011-9014.
14. H.-L. Wang, J.-M. Yan, S.-J. Li, X.-W. Zhang, Q. Jiang, *J. Mater. Chem. A* **2015**, 3, 121-124.
15. Q. Shi, D.-X. Zhang, H. Yin, Y.-P. Qiu, L.-L. Zhou, C. Chen, H. Wu, P. Wang, *ACS Sustainable Chem. Eng.* **2020**, 8, 5595-5603.
16. W. Kang, A. Varma, *App. Catal. B* **2018**, 220, 409-416.
17. K. V. Manukyana, A. Cross, S. Rouvimov, J. Miller, A. S. Mukasyana, E. E. Wolf, *Appl. Catal. A Gen.* **2014**, 476, 47-53.
18. J. He, Y. Qiu, S. Qin, P. Wang, *J. Mater. Chem. A* **2024**, 12, 32022-32029.
19. L. He, B. Liang, L. Li, X. Yang, Y. Huang, A. Wang, X. Wang, T. Zhang, *ACS Catal.* **2015**, 5, 1623-1628.
20. H. Wang, L. Wu, A. Jia, X. Li, Z. Shi, M. Duan, Y. Wang, *Chem. Eng. J.* **2018**, 332, 637-646.
21. H Zou, F. Guo, M. Luo, Q. Yao, Z.-H. Lu, *Int. J. Hydrogen Energy* **2020**, 45, 11641-11650.
22. X. Hong, Q. Yao, J. Long, X. Li, X. Chen, Z.-H. Lu, *Ind. Eng. Chem. Res.* **2021**, 60, 16224-16232.
23. M. Huang, Q. Yao, F. Guo, H. Zou, Z.-H. Lu, *Inorg. Chem.* **2020**, 59, 5781-5790.
24. Y. Liu, X. Liu, X. Liu, Y. Li, J. Ma, C. Ma, *Chemosphere* **2023**, 313, 137608.
25. X. Zhang, Q. Yao, H. Wu, Y. Zhou, M. Zhu, Z.-H. Lu, *App. Catal. B* **2023**, 339, 123153.
